# Egg-laying decisions based on olfactory cues enhance offspring fitness in *Stomoxys calcitrans* L. (Diptera: Muscidae)

**DOI:** 10.1038/s41598-019-40479-9

**Published:** 2019-03-07

**Authors:** Steve B. S. Baleba, Baldwyn Torto, Daniel Masiga, Christopher W. Weldon, Merid N. Getahun

**Affiliations:** 1 International Centre of Insect Physiology and Ecology (icipe), P.O. Box 30772-00100, Nairobi, Kenya; 2 Department of Zoology and Entomology, University of Pretoria, Private Bag X20, Hatfield, 0028 South Africa

## Abstract

Selection of oviposition substrate is critical in holometabolous insects. Female stable flies, *Stomoxys calcitrans*, locate and select vertebrate herbivore dung in which they lay their eggs. However, the preference for vertebrate herbivore dung by *S*. *calcitrans* females, its fitness consequences for offspring, and the semiochemicals used to locate and select oviposition substrates remain unclear. Using oviposition choice tests and life table bioassays we found that gravid female *S*. *calcitrans* prefer to oviposit on donkey and sheep dung, which also improves the performance of their offspring. GC-MS analysis followed by random forest classification identified β-citronellene and carvone as the most important predictive volatile organic compounds of donkey and sheep dung, respectively. In multiple choice oviposition bioassays, *S*. *calcitrans* laid more eggs in wet sand containing β-citronellene and carvone than in other treatments. The attractiveness of these compounds was confirmed in a field trial, with traps baited with β-citronellene and carvone catching more *S*. *calcitrans*. We conclude that gravid female *S*. *calcitrans* use semiochemical cues to choose oviposition substrates that maximise offspring fitness.

## Introduction

In holometabolous insects, oviposition site selection by gravid females plays an important role in their distribution, abundance, and population dynamics owing to the immobility of egg stages combined with the lack of parental care^1^. Location and selection of the most appropriate substrate for oviposition involves visual, olfactory and mechanical cues^2^. According to Städler^3^, the nutritional and chemical composition of the environment determine the success of development in almost all insects, thus olfactory cues play a paramount role during oviposition site selection. When selecting a suitable site for oviposition, insects might use a single chemical cue^4,5^ or a mixture of important chemical cues^6–9^.

Jaenike^10^ postulated that gravid female insects prefer to oviposit on substrate that maximise the fitness of their offspring. This was termed the ‘preference-performance’ or ‘mother knows best’ hypothesis. Oviposition substrates used by female insects include animal dung, fruits, and leaves which are abundant and rich in nutrients^4–6^. The preference-performance relationship has been widely explored in phytophagous insects, with some studies confirming a positive relationship between preference and performance^7–9^, while others show poor correspondence^11–15^. In hematophagous insects, understanding of the preference-performance relationship is most advanced in mosquitoes. For instance, there is a positive correlation between oviposition preference and larval performance in *Culiseta longiareolata*^16^, *Aedes triseriatus*, and *Aedes albopictus*^17^. whereas in *Wyeomyia smithii*^18^ and *Aedes aegypti*^19^, a negative correlation has been observed. However, these studies do not consider the chemical basis driving the preference-performance interaction. Here we investigated the preference-performance hypothesis in the stable fly, *Stomoxys calcitrans* (Diptera: Muscidae), and the chemical basis involved in this interaction.

*Stomoxys calcitrans* is a cosmopolitan blood-feeding insect of medical and veterinary importance^20^. For the success of their mating, egg production and survival, both female and male *S*. *calcitrans* depend on repeated blood meals from diverse domestic (e.g., camel, cattle, horse) and wild animal hosts (e.g., buffalo, antelope, zebra)^21,22^ as well as humans^23^. During their blood meals, *S*. *calcitrans* can transmit viruses (e.g., West Nile fever virus)^24^, bacteria (e.g. *Bacillus anthracis*)^25^, protozoans (e.g. *Trypanosoma evansi)*^26^ and helminths (e.g. *Habronema microstoma)*^27^. In the USA alone, Taylor *et al*.^28^ estimated that economic losses attributed to *S*. *calcitrans* infestation were >$2 billion per year.

The dung of vertebrate herbivore animals is used as an oviposition substrate by female *S*. *calcitrans*^29–31^. However, preference by *S*. *calcitrans* for vertebrate herbivore dung of different species, the fitness costs to its offspring, and the semiochemicals involved remain unclear. In this study, we investigated the preference-performance hypothesis in *S*. *calcitrans* oviposition behaviour on dung of different vertebrate herbivores, and the semiochemical basis of this interaction. We demonstrate that gravid female stable flies oviposit on substrates that have fitness benefits for their offspring using finely tuned responses to semiochemical cues in preferred substrates such as β- citronellene and carvone. We then used these semiochemicals to perform laboratory and field trials to test their attractiveness to female *S*. *calcitrans*. This information will prove useful for developing effective lures to attract and kill gravid females, and thereby suppress *S*. *calcitrans* abundance.

## Results

### Gravid female *S. calcitrans* prefer donkey and sheep dung for oviposition

**Figure 1:**
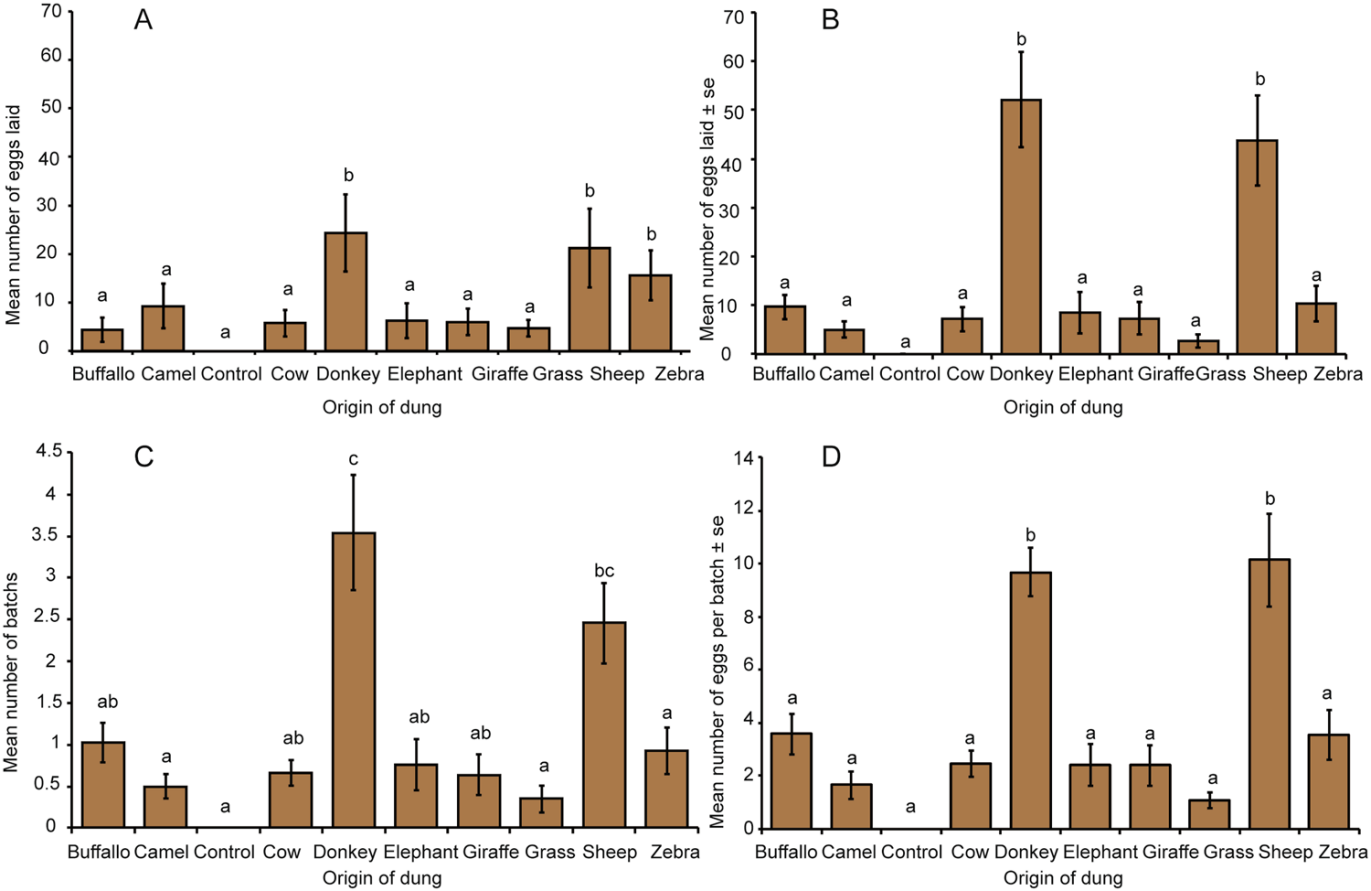
Gravid female *S*. *calcitrans* prefer to oviposit on donkey and sheep dung. (**A**) Mean number of eggs laid on dung of each vertebrate herbivore by wild female *S*. *calcitrans*. (**B**) Mean number of eggs laid on dung of each vertebrate herbivore by naïve female *S*. *calcitrans*. (**C**) Mean number of egg batches deposited on dung of each vertebrate herbivore by naïve female *S*. *calcitrans*. (**D**) Mean number of eggs per batch laid by naïve female *S*. *calcitrans*. Error bars indicate standard error of the mean (SEM). Bars with different letters are significantly different from each other (Kruskal-Wallis test followed by Dunn’s post hoc test; *P* < 0.05, n = 10).

Gravid female *S*. *calcitrans* consistently chose donkey and sheep dung as oviposition substrates over the other dung tested. The mean number of eggs laid on each substrate by gravid females was significantly different (Fig. 1A, Kruskal-Wallis test: H = 30.702, d.f = 9, *P* < 0.001). Females laid more eggs on donkey and sheep dung followed by zebra dung than on the dung of cow, camel, buffalo, elephant, or giraffe, grass and wet sand (control). Results obtained in the laboratory with naïve gravid female flies (7–10 days old) mirrored the results obtained in the semi-field assays (Fig. 1B; H = 59.497, d.f = 9, *P* < 0.001). The higher number of eggs laid on donkey and sheep dung resulted from a higher mean number of batches (Fig. 1C; H = 54.13, d.f = 9, *P* < 0.001) and mean number of eggs per batch (Fig. 1D; H = 59.38, d.f = 9, *P* < 0.001).

### Preferred oviposition substrates enhance fitness of *S*. *calcitrans* offspring

**Figure 2:**
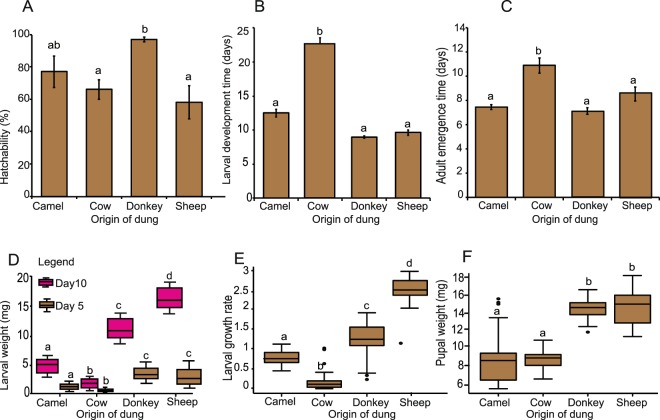
Gravid female *S*. *calcitrans* prefer to oviposit on substrates that enhance the fitness of offspring. (**A–C**) Bar plots showing: (**A**) egg hatchability, (**B**) larval development time, (**C**) adult emergence times when *S*. *calcitrans* offspring were reared in camel, cow, donkey, and sheep dung. Error bars represent SEM. Bars with different letters are significantly different from each other (ANOVA followed by SNK post hoc test; *P* < 0.05, n = 10). (**D–F**) Boxplots illustrating: (**D**) larval weight at five (brown) and ten days (pink), (**E**) larval growth rate, (**F**) pupal weight of *S*. *calcitrans* when raised in camel, cow, donkey, and sheep dung. Boxplot whiskers indicate ± 1.5 interquartile range limits. Box plots with different letters are significantly different from each other [grouped by the Kruskal-Wallis test followed by Dunn’s post hoc test for larval weight and larval growth rate, and ANOVA followed by SNK’s post hoc test for the pupal weight (*P* < 0.05, n = 10)].

Having shown that gravid female *S*. *calcitrans* prefer to oviposit in the dung of particular vertebrate herbivores, we tested whether female preference led to improved performance by offspring. Egg hatchability, larval and pupal development time, larval weight and pupal weight were recorded as fitness parameters. We found that egg hatchability (GLM, χ^2^ = 18.355, d.f = 3, *P* < 0.001), larval development time (F_3,36_ = 132.2, *P* < 0.001), larval weight (Day 5: H = 130.52, d.f = 3, *P* < 0.001; Day 10: H = 147.26, d.f = 3, *P* < 0.001), pupal development time or adult emergence (F_3,36_ = 17.48, *P* < 0.001), larval growth rate (H = 136.41, d.f = 3, *P* < 0.001), pupal weight (F_3,156_ = 140.4, *P* < 0.001) and adult emergence time (F_3,36_ = 17.48, *P* < 0.001) were affected by dung type. Egg hatchability was highest in donkey followed by camel, cow and sheep dung (Fig. 2A). Development time from egg to pupal stage was significantly shorter in donkey and sheep dung than in cow and camel dung (Fig. 2B). Larvae that developed in donkey and sheep dung were heavier at days 5 and 10 (Fig. 2D); consequently, they had the best growth rates (Fig. 2E). Pupae from donkey and sheep dung weighed more than those from cow and camel dung (Fig. 2F).

### Preference-performance relationship positively correlated with the physicochemical composition of preferred substrates

**Figure 3:**
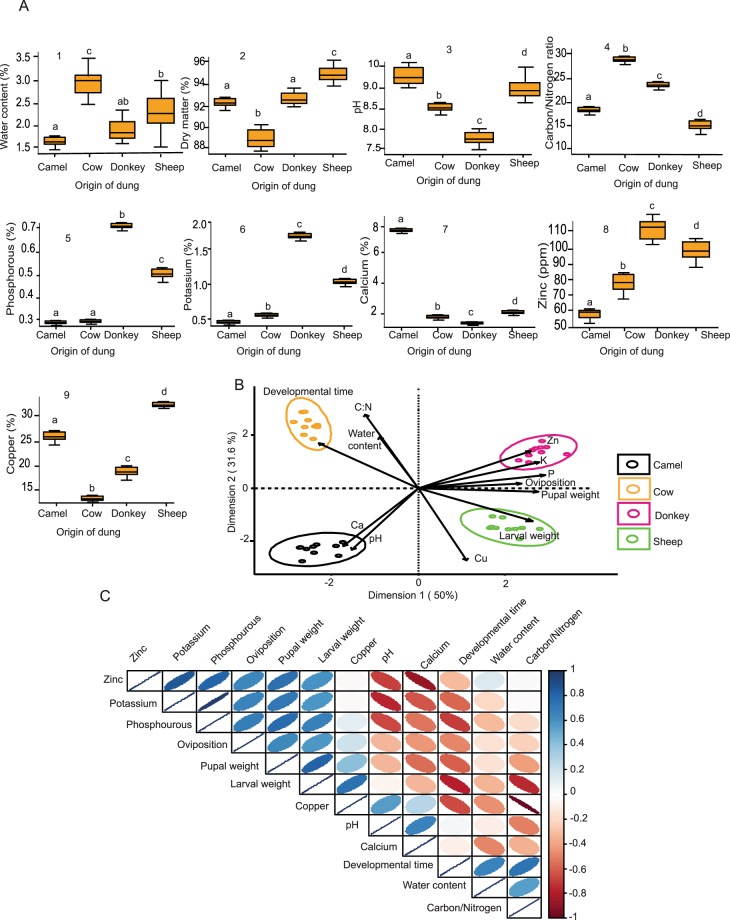
Oviposition substrates vary in their physiochemical properties. (**A**) Boxplot depicting results of physicochemical analysis of camel, cow, donkey and sheep dung: (1) water content, (2) dry matter, (3) pH, (4) carbon/nitrogen ratio, (5) phosphorus proportion, (6) potassium proportion, (**7**) calcium proportion, (8) zinc proportion and (9) copper proportion. The ends of boxplot whiskers represent the minimum and maximum of all the data. Boxes with different letters are significantly different from each other based on MANOVA followed by SNK post-hoc tests. (**B**) Principal component biplot showing the relation between *S*. *calcitrans* oviposition preference, larval performance and dung composition. Black ellipse: camel dung; orange ellipse: cow dung; magenta ellipse: donkey dung; and green ellipse: sheep dung. (**C**) Correlogram highlighting the direction and intensity of the correlation between oviposition preference, larval performance traits, and dung composition. Red and blue denote high negative and positive correlation, respectively; white indicates absence of correlation.

Our results clearly demonstrated that oviposition preference of gravid female *S*. *calcitrans* contributes to fitness of their offspring. Due to this, we set out to determine whether the preference-performance relationship was correlated with the physicochemical composition of the different oviposition substrates. We analysed the physical properties and micronutrient contents of the two preferred substrates (donkey and sheep dung) and non-preferred substrates (camel and cow dung). There was a significant difference in the physiochemical properties of the different animal dung (Fig. 3A, MANOVA: Pillai’s trace = 2.99, F_3,36_ = 2734, *P* < 0.001). Cow dung had the highest percentage of water content (Fig. 3A.1) and the lowest percentage of dry matter (Fig. 3A.2). The pH was lowest in donkey dung (Fig. 3A.3). We found a lower carbon/nitrogen ratio (Fig. 3A.4) and a higher amount of copper (Fig. 3A.9) in sheep dung. Phosphorus (Fig. 3A.5), potassium (Fig. 3A.6) and zinc (Fig. 3A.8), were higher in donkey and sheep dung; while calcium was highest in camel dung (Fig. 3A.7).

To correlate oviposition choice, fitness and physicochemical parameters, we performed a principal component analysis (PCA). We found these parameters to be highly correlated (Fig. 3B), indicating that *S*. *calcitrans* gravid females appear to consider the physiochemical properties of substrates when deciding to oviposit. The first two dimensions of the PCA explained 81.6% of the total variation. Dimension 1 was associated with the proportion of phosphorus and potassium in dung, and accounted for 50% of the total variation. Dimension 2, which accounted for 31.6% of the total variation, was highly correlated with carbon/nitrogen ratio and the proportion of copper in dung. To clearly illustrate correlations among measured variables, we constructed a correlogram (Fig. 3C). Oviposition was positively correlated with larval weight (r = 0.58, *P* < 0.001) and pupal weight (r = 0.64, *P* < 0.001); and negatively correlated with larval developmental time (r = −0.48, *P* < 0.001). Larval weight and pupal weight were positively correlated with the proportion of zinc (r = 0.59, *P* < 0.001; r = 0.75, *P* < 0.001), potassium (r = 0.59, *P* < 0.001; r = 0.73, *P* < 0.001), phosphorus (r = 0.68, *P* < 0.001; r = 0.77, *P* < 0.001), and copper (r = 0.75, *P* < 0.001; r = 0.42, *P* = *0*.*0074*) which were more abundant in preferred oviposition substrates. Larval weight and pupal weight were negatively correlated with the proportion of calcium (r = −032, *P* = 0.0436; r = −0.55, *P* < 0.001) and carbon/nitrogen ratio (r = −0.74, *P* < 0.001; r = −0.42, *P* = *0*.*0074*), which were higher in non-preferred oviposition substrates. Larval developmental time was positively correlated with dung water content (r = 0.66, *P* < 0.001) and carbon/nitrogen ratio (r = 0.73, *P* < 0.001), and negatively correlated with the proportion of phosphorus (r = 0.69, *P* < 0.001), potassium (r = 0.66, *P* < 0.001), and copper (r = −0.66, *P* < 0.001), which were again higher in donkey and sheep dung.

### Oviposition substrates are distinct in their volatile organic compound (VOC) composition

**Figure 4:**
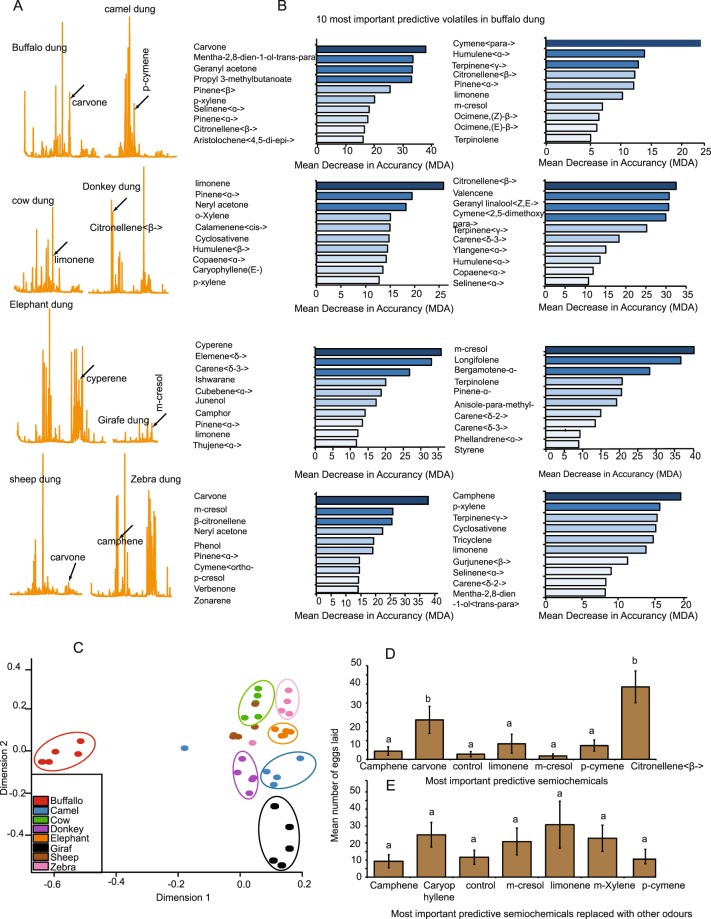
The most important volatiles from donkey (β-citronellene) and sheep (carvone) dung elicit the strongest oviposition response by *S*. *calcitrans* gravid females. (**A**) Representative GC–MS chromatogram of each vertebrate herbivore dung and most important semiochemicals. (**B**) Multidimensional scaling (MDS) plot showing the segregation of vertebrate herbivore dung based on their VOC composition. (**C**) Histogram showing the classification of the ten most important VOCs from vertebrate herbivore dung based on the Mean Decrease in Accuracy (MDA) of the Random Forest analysis. VOC associated with the darkest histogram has the highest MDA value and consequently, the most important. (**D**,**E**) Bar plots representing: (**D**) mean number of eggs laid by gravid female *S*. *calcitrans* on each oviposition medium (wet sand) loaded with the most important VOC of each vertebrate herbivore dung. Error bars represent SEM. (**E**) mean number of eggs laid by gravid female *S*. *calcitrans* on each oviposition medium (wet sand) loaded with the most important chemical volatile of each vertebrate herbivore dung with the replacement of β-citronellene and carvone by β-caryophyllene and m-xylene, respectively. Bars with different letters are significantly different from each other (Kruskal-Wallis test followed by Dunn’s post hoc test; *P* < *0*.*05*). Error bars represent SEM.

We determined whether oviposition preference behaviour observed in gravid female *S*. *calcitrans* (Fig. 1) was potentially mediated by olfactory cues by using coupled gas chromatography-mass spectrometry (GC-MS) to analyse the VOC composition of all dung types used in the oviposition bioassays. GC-MS analysis identified a wide range of VOCs emitted by the substrates (Fig. 4A). Using multidimensional scaling ordination, we found that each dung type had a distinct VOC composition (Fig. 4C).

### Signature VOCs of donkey and sheep dung elicit strongest oviposition

We hypothesised that gravid females of *S*. *calcitrans* use signature VOCs to locate suitable oviposition substrates, and that in our study these signature VOCs are represented by compounds that are abundant and permanently present (most important volatiles) in donkey and sheep dung. To test this hypothesis, we first classified the VOCs of each dung using a classification algorithm method called “random forest” (RF), which has been used successfully in others studies^32–34^. Based on the function *“importance ()”* embedded in the Random Forest R software package, we obtained the mean decrease in accuracy (MDA) of each volatile present in each dung. As the rule of thumb, VOCs with the highest MDA are, the most important, namely abundant and permanently present across all the replicates of tested vertebrate herbivore dung. Based on that rule we found that carvone was the most important VOC of buffalo and sheep dung; while *p*-cymene, limonene, β-citronellene, cyperene, *m*-cresol, and camphene were the most important VOCs of camel, cow, donkey, elephant, giraffe, and zebra dung, respectively (Fig. 4B), with a classification accuracy of 87.9%.

Having determined the most important VOC of each dung, we next asked if the most important VOC from donkey and sheep dung would stimulate more gravid female *S*. *calcitrans* to oviposit. To test this, we conducted a multiple-choice oviposition bioassay where gravid females of *S*. *calcitrans* were presented with wet sand loaded with the synthetic standard of the single most important VOC of each substrate at 10^−2^ v/v dilution. We found that oviposition by *S*. *calcitrans* varied across the tested VOCs (Fig. 4D: H = 25.30, df = 6, *P* < 0.001). In comparison with other media, gravid female *S*. *calcitrans* laid more eggs on the media loaded with β-citronellene and carvone, which are the most important VOCs of preferred oviposition substrates, donkey and sheep dung, respectively. To verify whether only β-citronellene and carvone led to the observed increase in number of eggs laid, we replaced β-citronellene and carvone with β-caryophyllene and *m*-xylene, which are the most important VOCs from donkey and sheep dung, respectively, and recorded oviposition. Neither β-caryophyllene nor *m*-xylene induced a significant increase in the mean number of eggs laid (Fig. 4E: H = 3.78, df = 6, *P* = 0.71). We concluded that β-citronellene and carvone were signature VOCs used by gravid females as olfactory cues to identify the best oviposition substrates.

### β- Citronellene and carvone enhance trap catch of *S*. *calcitrans*

**Figure 5:**
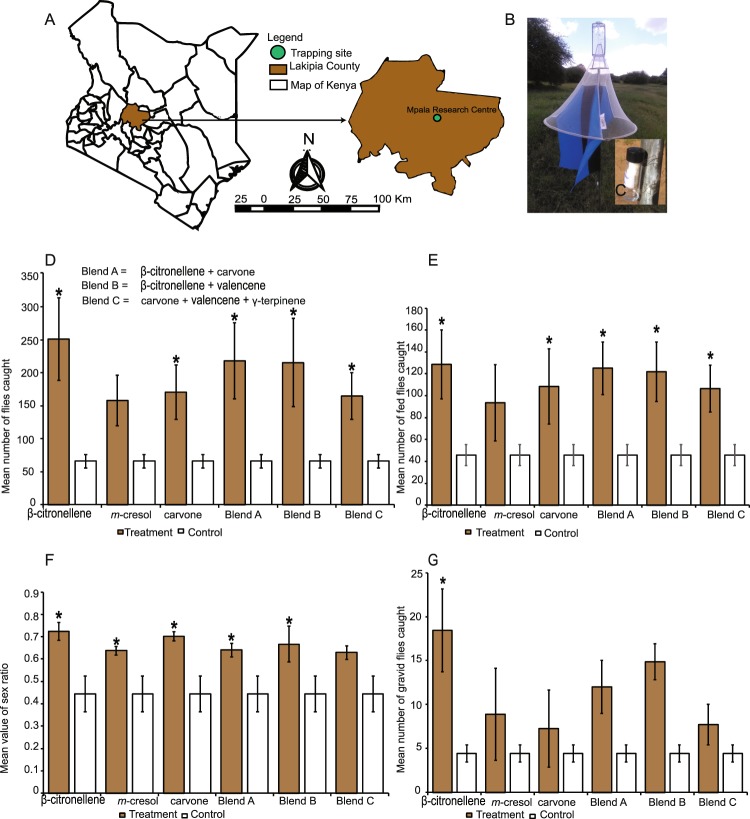
β-citronellene and carvone significantly enhance *S*. *calcitrans* trap catch. (**A**)Trapping site map, (**B**) Monoconical trap (**C**) Cotton roll dispenser in 4 ml vial with perforated cap. (**D–G**) bar plots depicting: (**D**) the mean number of *S*.*calcitrans* caught, (**E**) the mean number of *S*.*calcitrans* blood fed flies caught, (**F**) the mean value of *S*.*calcitrans* sex ratio, (**G**) the mean number of *S*.*calcitrans* gravid females caught. Error bars represent SEM., treatment with an asterisk (*) above the error bar are significantly different from the negative control (unbaited trap) [Dunnett’s t-test (P < 0.05, n = 7)].

Having identified the VOCs that stimulate gravid female *S*. *calcitrans* to oviposit more on donkey and sheep dung, we assessed their attractiveness under field conditions. A Latin square design experiment was performed at Mpala Research Centre (www.mpala.org) located in Laikipia County, Kenya (Fig. 5A) using monoconical traps (Fig. 5B). Traps were baited with different VOCs (Fig. 5C) and rotated daily to account for any bias resulting from trap location^35^. Each treatment consisted of an undiluted solution of β-citronellene, carvone, Blend A (carvone + β-citronellene), Blend B (β-citronellene + valencene), Blend C (carvone + valencene + γ- terpinene), *m*-cresol (positive control: already known to attract *S*. *calcitrans*; see Tangtrakulwanich^36^), or a negative control (unbaited trap).

Overall, we caught more *S*. *calcitrans* [8702 (85%)] than other insects [1739 (15%)] (χ^2^ = 4643.6, df = 1, p < 0.001). Other insects mainly comprised house flies, *Musca domestica* [1706 (98.1%)]. The number of *S*. *calcitrans* caught significantly varied with VOC treatment (GLM.nb: LR = 15.74, df = 6, *P* < 0.05). The mean number of *S*. *calcitrans* caught by traps baited with β-citronellene, Blend A, Blend B, carvone, Blend C and *m*-cresol were respectively 3.9, 3.3, 3.30, 2.6, 2.5 and 2.4 times more than those caught in unbaited traps (Fig. 5D).

Irrespective of VOC treatment, we caught significantly more blood fed (58.6%)) than unfed *S*. *calcitrans* (41.41%) (χ^2^ = 256.5, df = 1, *P* < 0.001). The number of blood-fed flies varied significantly among VOC treatments (GLM.nb: LR = 9.83, df = 6, P < 0.05). Traps baited with β-citronellene, Blend A, Blend B, carvone and Blend C caught significantly more blood-fed *S*. *calcitrans* than the unbaited trap. Conversely, trap catches of blood-fed *S*. *calcitrans* with *m*-cresol baited and unbaited traps were not significantly different (Fig. 5E).

During the entire period of trapping, we caught significantly more female *S*. *calcitrans* (65%) than males (35%) (χ^2^ = 741.39, df = 1, *P* < 0.001). The VOC treatment significantly affected the sex ratio (female to total number of individuals ratio) of caught flies (F_6,42_ = 3.74, *P* = 0.0045). The sex-ratio was female-biased in traps baited with β-citronellene, carvone, Blend B, Blend A, and *m*-cresol, while the unbaited traps caught the same number of females and males (Fig. 5F).

The number of gravid females caught significantly differed among the VOC treatments (GLM.nb: LR = 14.35, df = 6, *P* < 0.05). Among all the VOC treatments, only traps baited with β-citronellene caught significantly more gravid females of *S*. *calcitrans* than the unbaited traps. The mean number of gravid flies caught by traps baited with carvone, *m*-cresol, Blend A, Blend B, Blend C was not significantly different from the unbaited trap catch (Fig. 5G).

## Discussion

Our results demonstrate that gravid female *S*. *calcitrans* exhibit a preference-performance oviposition behaviour that is mediated by olfactory cues. Additionally, these results demonstrate that oviposition preference is associated with better fitness of *S*. *calcitrans* immature stages.

Results from the oviposition preference bioassay revealed that gravid female *S*. *calcitrans* preferred to lay eggs on donkey dung followed by sheep dung. This has also been reported by Hafez & Gamal-Eddin^30^ but the causes for this preference and the fitness benefits were not investigated. We demonstrated that oviposition preference by female *S*. *calcitrans* was related to better fitness of *S*. *calcitrans* immature stages. For instance, larvae and pupae of *S*. *calcitrans* were heavier and developed more rapidly on donkey and sheep dung. This may reflect the higher nutritional value of these specific substrates for *S*. *calcitrans*^37^. We show that nitrogen, zinc, potassium, phosphorous and copper content were significantly higher in donkey and sheep dung. These elements were positively correlated with larval and pupal weights and negatively correlated with developmental time. The positive effect of these elements in insect fitness is widely acknowledged in the literature^38–41^. Similarly, Perkins *et al*.^42^ demonstrated that augmentation of the diet with phosphorus increased the growth rate of the tobacco hornworm, *Manduca sexta* (L) (Lepidoptera: Sphingidae).

Evidence for the preference-performance relationship has been demonstrated in other insect groups although studies have been heavily biased towards herbivorous insects. For example, Heisswolf *et al*.^43^ found that females of the monophagous beetle *Cassida canaliculata* (Coleoptera: Chrysomelidae) preferred to oviposit on larger host plants (rich in nitrogen), which then led to improved larval performance and survival in comparison with larvae developing on smaller host plants. Similarly, Chen *et al*.^44^ have shown that cotton with a higher level of nitrogen is suitable for oviposition and development of larvae of the beet armyworm, *Spodoptera exigua* (Hübner) (Lepidoptera: Noctuidae). When offered a choice between two cultivars of *Brassica oleracea* (Derby Day and Drago), female diamondback moths, *Plutella xylostella* (Lepidoptera: Plutellidae) laid more eggs on the cultivar with lower glucosinolate concentration, which also maximised larval performance^45^.

Our results revealed that the preference-performance behaviour observed in *S*. *calcitrans* was driven by olfactory cues. We demonstrated that although vertebrate herbivore dung contained a plethora of VOCs [more than 45 each (Fig. 4A)], *S*. *calcitrans* used a single semiochemical to select an oviposition site. For instance, only one VOC identified using random forest analysis was enough to mimic the presence of preferred or unpreferred vertebrate herbivore dung. As predicted, the most important VOC from donkey dung (β-citronellene) was enough to mimic the stimulatory effect of the substrate. On the other hand, media loaded with carvone did not significantly differ from the other media (Fig. 5D). This result shows that to mimic the response of gravid *S*. *calcitrans* to sheep dung, some components might be missing. Several studies report that a single compound might be enough for females to detect oviposition substrates and initiate oviposition^4,46^, while in other cases a signature blend of VOCs might be required for the same outcome^6,47^. In the absence of β-citronellene and carvone, *S*. *calcitrans* gravid females failed to exhibit a preference for any substrate. Instead, they laid eggs randomly on each presented medium including those that led to poor fitness, demonstrating the importance of β-citronellene and carvone in oviposition-site selection. However, it is not clear how these semiochemicals relate to the nutrient value of the substrates. It is likely that other sensory modalities play a role, such as taste. Jeanbourquin & Guerin^29^ reported the presence of β-citronellene in horse dung (like donkey, in the family Equidae) with electrophysiological activity in *S*. *calcitrans* antennae, but the authors did not go on to explore the effect of this chemical on *S*. *calcitrans* behaviour.

Synthetic semiochemicals eliciting a specific behaviour in insects can enhance insect catches when used as a bait in field trapping systems^48–50^. This was confirmed in our field work when we baited monoconical traps with β-citronellene, carvone, Blend A, Blend B, and Blend C. In this experiment, *S*. *calcitrans* represented 85% of insects caught, which supports the results of Mihok *et al*.^51^ and Tunnakundacha *et al*.^52^ who reported that monoconical traps are efficient for trapping flies in the subfamily Stomoxyinae. Traps baited with β-citronellene, carvone, Blend A, Blend B, and Blend C captured more flies than unbaited traps (negative control) and traps baited with *m*-cresol (positive control) (Fig. 5D). When checked for feeding status, traps baited with the same VOC treatments captured more engorged flies than the unbaited traps (Fig. 5E). A possible explanation for this result is that vertebrate herbivore dung is more likely to be visited by fed females for oviposition. Additionally, males of *S*. *calcitrans* are also attracted to dung-derived semiochemicals, most probably to look for females for mating. Interestingly, when we checked for gravid status, we found that only traps baited with β-citronellene caught significantly (4.5 times) more gravid female *S*. *calcitrans* than unbaited traps. Also, traps baited with blends containing β-citronellene [Blend A (carvone + β-citronellene) and Blend B (β-citronellene + valencene)] captured more gravid flies than traps baited with carvone, *m*-cresol, and Blend C (carvone + valencene + γ-terpinene) (Fig. 5G). This clearly shows that, either as a single or combined with other dung volatiles, β-citronellene efficiently attracts gravid female *S*. *calcitrans*.

In conclusion, gravid female *S*. *calcitrans* exhibit a preference-performance oviposition behaviour driven by signature odours emanating from vertebrate herbivore dung. Larvae and pupae developing from eggs laid on preferred substrates exhibit higher fitness than those that develop on non-preferred substrates. Furthermore, a single or blend of signature VOCs such as β-citronellene and carvone increased *S*. *calcitrans* female attraction and stimulated oviposition both under laboratory and field conditions. The high level of female-biased attraction, including high levels of gravid female and blood-fed flies, to the semiochemical β–citronellene associated with oviposition substrates, is promising for its potential use in the management of *S*.*calcitrans*.

## Material and Methods

### Insects and oviposition substrates

To establish the colony, wild individuals of *S*. *calcitrans* were captured at *icipe* Duduville campus in Nairobi (1°13′12″S, 36°52′48″E; 1,600 m above sea level) using a Vavoua trap. Trapped adults were transferred to cages (75 × 60 × 45 cm) in an insectary maintained at 25 ± 5 °C and 65 ± 5% relative humidity, with a 12 L: 12 D photocycle. Flies were fed two times per day (0800 and 1600 hours) on defibrinated bovine blood on moistened cotton. The rearing medium consisted of rabbit dung. Rabbit dung was placed in plastic containers (21.5 × 14.5 × 7.4 cm) that were introduced to the adult cage for two days to allow oviposition. Afterwards, the rearing medium was removed and transferred to another cage and followed daily from egg hatch to the pupal stage. Pupae were placed in Petri dishes, removed from the cage, and introduced to another cage for age-matched adult emergence. As described above, blood and rearing media were provided to the newly emerged adults to obtain flies for experiments.

For our oviposition bioassay, we screened fresh dung of buffalo, camel, cow, donkey, elephant, giraffe, sheep, and zebra, which are potential breeding sites for *S*. *calcitrans* and abundant in the region where the study was conducted. The dung types were collected from different agroecological zones, including Kapiti Plain in Machiakos County (1°37′60″S, 37°0′0″E), Ngurunit in Marsabit County (1°59′58″S, 37°30′11″E), and Shimba Hills located in Kwale County (04°15′26″S, 39°23′16″E), Kenya. These localities are characterised by the presence of several populations of wild and domestic vertebrate herbivores, as well as biting flies such as *S*. *calcitrans*. Dung was collected immediately after it was deposited by each species (within 24 hours). To our knowledge, none of the vertebrate herbivore populations from which dung was collected received anthelmintic treatments that could potentially affect development of coprophagous insects^53^.

### Preference-performance hypothesis test

#### Multiple-choice oviposition preference bioassay with vertebrate herbivore dung

To assess oviposition preference of gravid female *S*. *calcitrans*, we began with a multiple-choice oviposition bioassay in semi-field conditions, using wild, gravid female, easily recognized by examining the ventral abdomen filled with eggs. *S*. *calcitrans* caught directly from the field. A cage (75 × 60 × 45 cm) containing 30 wild gravid females was placed outside in a shaded, sheltered location. Fresh dung (60 g) from buffalo, camel, cow, donkey, elephant, giraffe, sheep, and zebra, as well as grass and wet sand (control), was placed in Petri dishes and introduced to the cage. Each Petri dish was separated by at least 20 cm in a circle, with the order of dung being haphazard. Twenty-four hours after setting up the bioassay, the total number of eggs laid on each substrate was counted. The cages were placed outside at the *icipe* Nairobi campus from May to June 2017, during which 30 replicates were performed. Mean temperature during this period was 22.5 ± 4.7 °C, with 55% relative humidity, and 12 L: 12 D photoperiod.

To check the validity of the result from field collected flies under semi-field conditions, we conducted the same experiment in a controlled laboratory environment (at 25 ± 5 °C and 65 ± 5% relative humidity, with a 12 L:12 D photocycle) using 30 naïve, gravid females *S*. *calcitrans* (aged from 4 to 6 days) from our established culture. Here, the oviposition preference was assessed after 24 hours by recording three parameters: (1) number of batches deposited on each substrate, (2) number of eggs per batch, and (3) the total number of eggs deposited on each substrate. The laboratory experiment was replicated ten times.

### Performance test

We conducted an incomplete cohort life table study for the immature stages of *S*. *calcitrans* using camel, cow, donkey and sheep dung. This was to test if a detected preference of gravid female *S*. *calcitrans* to oviposit on donkey and sheep dung in comparison with camel and cow dung was related to the performance of their offspring. Ten *S*. *calcitrans* eggs were artificially introduced to each type of dung (n = 10) and followed daily until adulthood to record six parameters: (1) egg hatchability, (2) larval development time (from egg to pupa), (3) pupal development time (from pupa to adult eclosion), (4) larval weight, (5) larval growth rate and (6) pupal weight. Eggs for each treatment were obtained by placing the same dung type in the established culture cage 24 hours before commencing the test. The weight parameter was recorded individually on 40 larvae and pupae coming from each substrate. Larval weight was recorded 5 and 10 days after egg hatch. Larval growth rate was calculated as^13^ [M_day10_−M_day5_]/t, where M_day10_ was the larval mass at day ten, and M_day5_ was the larval mass at day five, and t, was the number of days intervening between the two consecutive weight measurements.

### Dung sample physico-chemical characterisation

To elucidate whether the performance of *S*. *cacitrans* offspring is related to the chemical composition of their substrate of development, we determined the proportion of nitrogen (N), carbon (C), pH, and micronutrients [copper (Cu), phosphorus (P), potassium (K), zinc (Zn)] present in camel, cow, donkey and sheep dung using lyophilised (freeze-dried), ground samples (n = 10). The total N was determined using the Kjeldahl method with 0.5 g of each dung material^54^. The total C expressed as a percentage of residues was determined after 4 hours of ignition at 500 °C in a muffle furnace using a 0.5 g sample of each dung^55^. The pH was measured using the potentiometric method after water sample extraction (by shaking 1:2 w/v of each sample for 20 minutes at 180 rpm). The micronutrients were measured from 0.5 g of each sample using the atomic emission spectrometry (ICP-OES) method following the microwave digestion procedure with nitric acid and hydrochloric acid^56^. Additionally, to determine the water content of each animal dung, we recorded the volume (V) and weight (W_wet_) of ten fresh samples. Afterwards, we determined the dry weight (W_dry_) of the same samples by placing them in an oven at 100 °C and reweighing them several times until constant weight. We calculated the water content, and the proportion of dried matter of each animal dung using the formula^54^ (W_wet_−W_dry_)/ W_dry_; and (W_wet_−W_dry_)/V.

### Characterisation of semiochemicals emanating from oviposition substrates

#### Odour collection

Odours were collected from fresh dung samples from buffalo, camel, cow, donkey, elephant, giraffe, sheep, and zebra using a dynamic headspace apparatus^57^. In this setup, ambient air was passed through copper tubing to activated charcoal (for purification), then into a bubble humidifier containing double distilled water. The humidified air was supplied by a vacuum at a flow rate of 150 ml air min^−1^ through multiple ports (manifolds) to the dung samples enclosed in glass jars, which were connected in parallel. Each glass jar had a port for a Super Q adsorbent, which trapped any volatile organic compounds from the vacuum air stream. For our collection, 500 g of each dung type (replicated five times) was introduced and sealed inside the glass jars (oven sterilised at 100 °C for 24 hours). Before use, each adsorbent was cleaned ten times with 200 µL hexane, followed by dichloromethane to avoid contamination. Volatiles were collected for 24 hours. Trapped volatile compounds were eluted by washing the adsorbent with 600 μl hexane and stored in air-tight glass vials at −20 °C until analysed by gas chromatography linked with mass spectrometry.

### Gas chromatography linked with mass spectrometry

The collected volatile compounds were analysed using a gas chromatograph coupled to a mass spectrometer (GC–MS; HP 6890 GC and 5975 MS; Agilent Technologies, Palo Alto, CA, USA) in the electron impact ionisation mode at 70 eV. Each sample (1 μL) was injected into the GC-MS with an autosampler (Agilent Technologies). Injections of the volatile extracts were conducted with a splitless injector at 220 °C. Compounds were separated on a nonpolar capillary HP column with helium as the carrier gas at an average linear flow rate of 35 cm s^−1^. The oven temperature was held at 35 °C for 5 min and then increased by 10 °C/min to a final temperature of 280 °C, which was held for 10 min. Volatiles were then identified by comparison of their mass spectra and retention times with those of commercial standards and library database spectra using the NIST mass spectral search program (ver. 2.0), Pherobase (http://www.pherobase.com) and the NIST web book (http://webbook.nist.gov/chemistry). The identified chemicals were confirmed by co-injection with authentic standards and comparison with both the expected retention time and MS spectra.

### Volatile chemical classification

To identify the volatiles that are abundant and permanently present across all the replicates of each herbivorous vertebrate dung (most important volatiles), we performed random forest (RF) analysis using the relative abundance of each identified volatile organic compound^58^ The RF analysis is a mathematical algorithm that uses results from several decisions trees to classify a large number of variables (chemical volatiles in our case)^59^. Compared to other classification methods such as principal component analysis (PCA), the RF analysis is advised for volatile importance classification. This is because (1) it allows for more variables; (2) it has a good classification efficiency; (3) it is capable of arriving at a minimal set of variables that can be used as predictors for a particular group; (4) it is robust to interactions and correlations among variables; (5) it gives measures of relative variable importance; and (6) it can also be used to analyse time series data that record patterns in volatile emissions over time^59–61^

### Multiple-choice oviposition bioassay with the most important dung volatiles

The RF analysis identified carvone as the most important volatile of buffalo and sheep dung. On the other hand, *p*-cymene, limonene, β-citronellene, cyperene, *m*-cresol, and camphene were identified as the most important volatiles of camel, cow, donkey, elephant, giraffe, and zebra dung, respectively. Subsequently, we purchased the synthetic standards of the following compounds: (R)-(−)-carvone (98%), *p*-cymene (99%), (R)-(+)-limonene (93%), (+)-β-citronellene (analytical standard), *m*-cresol (98%), and camphene (95%) (Sigma-Aldrich Germany). Cyperene was not tested due to its unavailability of a commercial product.

The attractiveness of each volatile for *S*. *calcitrans* oviposition was tested using the concentration 10^−2^ v/v diluted in mineral oil. For the bioassay, seven Petri dishes containing wet sand as an oviposition medium were introduced to a cage (75 × 60 × 45 cm). We placed an Eppendorf tube lid loaded with 100 µl of each volatile solution on the wet sand. The control was wet sand with mineral oil. Thereafter, 30 gravid females of *S*. *calcitrans* were introduced to the cage. After 24 hours the total number of eggs deposited on each medium was counted. The experiment was replicated 15 times. Wet sand (control) was avoided by gravid females of *S*. *calcitrans* in our earlier oviposition preference bioassays, so any enhancement of oviposition indicated a stimulant effect on females.

The results of this bioassay revealed that media containing carvone and β-citronellene from sheep and donkey dung, respectively, attracted more gravid females of *S*. *calcitrans* for oviposition over the other volatiles. To appreciate the attractiveness effect of carvone and β-citronellene in gravid female *S*. *calcitrans* oviposition, we conducted another oviposition preference bioassay by replacing these two volatiles with β-caryophyllene and *m*-xylene. β-caryophyllene and *m*-xylene were present in donkey and sheep dung respectively but less important. The same methods were used for this bioassay, which was replicated 15 times, and the total number of eggs laid in each Petri dish of wet sand was counted after 24 hours.

### Field trapping assay

Finally, we tested the attractiveness of carvone and β-citronellene to *S*. *calcitrans* (mainly gravid females) under field conditions. To do so, we conducted a field trapping study at Mpala Ranch located in Laikipia County in central Kenya (Fig. 5A; 00° 23′26.98″N, 036°52′14.98″E). This region is characterised by semi-arid savannah vegetation in which *S*. *calcitrans* are associated with wild animals (elephant, zebra, impala, monkey, lion etc.) and domestic animals (mainly camel, cattle, goat and sheep). Vavoua traps^21^; (Fig. 5B) were baited with 2 ml of undiluted synthetic standards of β-citronellene, carvone, and *m*-cresol, which was a positive control, already known to attract *S*. *calcitrans*^36^. Blends of volatile chemicals were also tested. These were formulated using the mean decrease accuracy (MDA) value of their chemicals. Blend A comprised carvone +β-citronellene (3:2), Blend B comprised β-citronellene + valencene (1:1), and Blend C comprised carvone + valencene + γ-terpinene (3.5:3.5:3). Each compound or blend was transferred to a 4 ml glass vial. A cotton dental roll (10 × 38 mm; Shanghai Dochem Industries Co. Ltd.) was inserted inside the vial as a dispenser. The vial was closed with a perforated cap (5 holes of 2 mm diameter^62^) and gently tilted to soak the cotton dispenser (Fig. 5C). Traps with vials without chemicals were used as a negative control. Each vial was fixed on the pole of each trap with a metal wire, 0.5 m above the ground.

Traps were placed 150 m apart in a Latin square design. Trapping was carried out for 7 days with daily rotation of vials among the traps. Seven traps per lure were set before 09:00 h daily and checked twice per day at 13:00 h and 17:00 h. *Stomoxys*. *calcitrans* were identified based on the key of Zumpt^63^. Identified individuals were sorted by sex, blood-feeding status and egg development. Feeding status and egg development were established by gently piercing the abdomen of each captured female with a needle to verify the presence of blood and eggs, respectively. Three parameters were therefore recorded for each trap: (1) the number of males and females of *S*. *calcitrans* caught, (2) the number of fed flies caught and (3) the number of gravid females caught.

### Data analysis

All analyses were performed using R software^64^ (version 3.5.1) and the R Studio graphical user interface (version 1.1.383). The data from the parameters we used to assess oviposition preference of gravid female *S*. *calcitrans* (number of batches deposited on each substrate, number of eggs per batch and the total number of eggs laid on each substrate) were subjected to the Shapiro-Wilk test of normality and Levene’s test of homoscedasticity. Data were not normally distributed, and variances were not homogeneous (p < 0.05). Therefore, we used the non-parametric Kruskal-Wallis test followed by Dunn’s post hoc test with Bonferroni’s adjustment (to avoid type I error) to determine whether *S*. *calcitrans* oviposition differed among the tested substrates^65^.

Immature performance parameters were analysed in relation to substrate as the dependent variable. Egg hatchability data were analysed using a generalised linear model (GLM) with binomial distribution due to the binary nature of this parameter (hatched vs unhatched)^66^. The number of hatched and unhatched eggs were taken as the response variable. Model significance was detected by analysis of deviance (with the chi-squared test). Tukey’s multiple comparisons tests were performed using the package ‘lsmeans’^67^ to identify differences in egg hatchability among substrates. Larval development and adult emergence time data were normally distributed (Shapiro-Wilk test: p > 0.05). so we used analysis of variance (ANOVA) followed by Student-Neuman-Keuls (SNK) post hoc multiple comparisons tests using the R software package called ‘Agricolae’^68^ to separate means from each substrate. Larval weight and larval growth rate data were analysed using the non-parametric Kruskal-Wallis test followed by post-hoc Dunn’s tests due to the non-normal distribution of the data and the disparity of their variance. For pupal weight, data were normally distributed, so we used ANOVA followed by the SNK post-hoc tests.

To compare the physicochemical composition of camel, cow, donkey, and sheep dung, we performed the multivariate analysis of variance (MANOVA) followed by SNK tests. To establish whether dung constituents were correlated to *S*. *calcitrans* oviposition preference and the performance of their offspring, we performed principal components analysis (PCA) using two R packages called “FactoMineR” and “Factoextra”^69^.

We classified the chemical volatiles arising from the dung of each vertebrate herbivore, using the R software package “RandomForest”^58^, version 4.6–12. To execute the RF analysis, we ran 10000 iterations (ntree) with 11 volatiles randomly selected at each split (mtry = √q, where q is the total number of volatiles (120)). Based on the function ‘*importance ()”* we generated the mean decrease in accuracy (MDA), which provides an importance score for each volatile. For each herbivore dung, the volatile with the highest MDA value was considered the most important^32,61^. To visualise the similarity of herbivore dung volatile composition, we generated a multidimensional scaling (MDS) ordination plot^70^ using the function “*MDSplot()”*of the “RandomForest” package.

Data from multiple-choice oviposition with important volatiles were analysed with the non-parametric Kruskal –Wallis test followed by the post hoc Dunn’s test.

For the results from the field trapping assay, we used a generalised linear model with negative binomial error distribution and log-link to determine whether bait enhanced *S*. *calcitrans* catches (package ‘MASS’^71^, function ‘glm.nb’). Post-hoc Dunnett’s tests (R function glht from the package multcomp) were used to compared the number of flies caught by each baited trap with the unbaited control trap^72^. We compared the following parameters: (1) the number of flies, (2) the number of fed flies, and (3) the number of gravid females caught by each trap. For the sex ratio parameter (female number/total number of flies), we used ANOVA followed by Dunnett’s test. All statistical results were considered significant when *P* < 0.05.

## Data Availability

The raw data generated and analysed in the current study are available from the corresponding author on reasonable request.
